# An Evaluation of the Levels of Vitamin D and Bone Turnover Markers After the Summer and Winter Periods in Polish Professional Soccer Players

**DOI:** 10.2478/hukin-2013-0053

**Published:** 2013-10-08

**Authors:** Aleksandra Kopeć, Krzysztof Solarz, Filip Majda, Małgorzata Słowińska-Lisowska, Marek Mędraś

**Affiliations:** 1 Department of the Biological Basis of Sport, University of Physical Education, Wrocław, Poland.; 2 4th Military Hospital in Wrocław, Poland.

**Keywords:** 25(OH)D3, bone metabolism, professional soccer players

## Abstract

Vitamin D is synthesised in the skin during exposure to sunlight. The fundamental roles of vitamin D are the regulation of calcium and phosphate metabolism and bone mineralisation. Low vitamin D levels in athletes may adversely affect their exercise capabilities. The aim of our study was to investigate changes in serum levels of 25(OH)D3, calcium and bone turnover markers in football players in two training periods differing in the exposure to sunlight (after the summer period and after the winter period). We investigated 24 Polish professional soccer players. Serum levels of the following parameters were determined: 25(OH)D3, calcium, osteocalcin (OC), parathormone (PTH), procollagen type I N - terminal peptide (P1NP), and beta - CrossLaps (beta - CTx). We showed significantly higher levels of 25(OH)D3 and calcium and lower levels of PTH after the summer period versus the winter period. No significant differences in the levels of bone turnover markers were found. Furthermore, we did not observe any significant correlations between the levels of 25(OH)D3 and other parameters. Normal levels of 25(OH)D3 were observed in 50% of the players after the summer period and only in 16.7% of the players after the winter period. It is justified to measure the levels of 25(OH)D3, calcium and PTH in soccer players, especially after the winter period, when the exposure to sunlight is limited.

## Introduction

Vitamin D is a lipophilic prohormone that may originate from the diet, although its principal source is endogenous synthesis of the vitamin in response to sunlight. Ultraviolet B (UVB) radiation causes the conversion of 7 dehydrocholesterol to provitamin D3. If the exposure to sunlight is adequate, provitamin D3 undergoes rapid conversion to vitamin D3 (cholecalciferol). Vitamin D3 is converted to 25(OH)D3 in the liver and 25(OH)D3 is subsequently converted to the active form - 1,25(OH)2D3, in the kidneys (Holick, 2007).

Vitamin D receptors have been identified in human cells of the bone and skeletal muscles, what may suggest its considerable role in bone mineralisation and the synthesis of muscle proteins (Bischoff - Ferrari et al., 2004a). The significance of vitamin D for the regulation of calcium and phosphate metabolism and for muscle function has been demonstrated by many authors (Hamilton, 2010). Low levels of vitamin D in athletes have been shown to decrease muscle strength and significantly increase the risk of bone and muscle injuries (Cannell et al., 2009; Willis et al., 2008).

Analyses conducted in various groups of athletes, such as runners (Lethonen - Veromaa et al., 1999; Willis et al., 2008), gymnasts (Lethonen - Veromaa et al., 1999; Lovell, 2008) or amateur athletes (Halliday et al., 2011) revealed that 37 – 100% of the subjects had 25(OH)D3 deficiency and 1 – 83% of the subjects had 25(OH)D3 insufficiency. These results depended on the sports discipline, study period and geographic location.

The aim of our study was to investigate serum levels of 25(OH)D3 and bone turnover markers in Polish professional soccer players after two training periods: after the summer and the winter period.

## Material and Methods

A total of 24 Caucasian Polish professional soccer players were included in the study. The mean age was 26.24±3.41 years. The players trained outdoors during the two investigated periods. Training took place twice a day, 2 – 3 hours each in Wrocław, Poland, which is situated at the latitude of 51°10′ N. The exercise loads during the two investigated training periods were very similar. In January, the players spent two weeks at a training camp in Cyprus (at the latitude of 30°11′ N) and trained outdoors. After – summer (September) the uniform covered 36% of body and after – winter (April) the uniform covered 80% of body.

The players’ diet in both periods was very similar. During the study period the subjects did not consume any food supplements containing vitamin D or calcium.

Body mass was measured with an electronic scale. Body composition (fat, fat - free body mass, water content and muscle mass) was evaluated with the bioelectric impedance analyser BIA manufactured by Akern Bioresearch (Italy). To assess the body area which was covered by uniform the rule of nines was used (Knaysi et al., 1968). Information about the diet was measured on the basis of food diaries.

Blood was sampled at 8.00 am after a 12 - hour fast and 24 hours without training. Blood for determination of after - summer values was sampled in September and that for determination of after - winter values was sampled in April. The serum samples were separated and stored at −70°C.

Serum levels of 25(OH)D3, osteocalcin (OC), parathormone (PTH), procollagen type I N -terminal peptide (P1NP), and beta - CrossLaps (beta - CTx) in the two training periods under investigation were determined by electrochemiluminescence (ECLIA) using the Elecsys system (Roche, Switzerland). For 25(OH)D3, the intra - and interassay coefficients of variation (CVs) were 5.6% and 8.0%, respectively, and the limit of detection was 4 ng/ml (10 nmol/l). The respective values for PTH were: 4.5%, 4.8% and 1.20 pg/ml (0.127 pmol/l), those for P1NP were 2.3%, 2.8% and <5 ng/ml, those for OC were 2.9%, 4.0% and <5 ng/ml, and those for beta - CTx were 2.5%, 3.5% and 0.01 ng/ml (10 pg/ml). Serum calcium was determined by colorimetry using the Konelab 60 system from bioMérieux (France). The intra - and interassay CVs were 1.4% and 1.95%, respectively, and the limit of detection was 0.36 mmol/l (1.4 mg/dl).

The study was approved by the Bioethics Committee of the University School of Physical Education in Wrocław, Poland.

### Statistical analysis

Statistical analyses were performed using PQStat for Windows (version 1.4.4.126). The changes between September and April in the levels of bone turnover markers were analysed with parametric tests (the t - Student test) for normally distributed variables and with non - parametric tests (the Wilcoxon signed - rank test) for variables that did not meet the criterion for normal distribution. The Spearman correlation coefficient was used to detect correlation. P values less than 0.05 were considered statistically significant.

## Results

The results of our study are summarised in Tables 1 and 2. There were no significant differences in the mean values of anthropometric parameters or body mass composition between the two investigated training periods (Table 1).

Table 2 summarises the mean and SD values for serum levels of 25(OH)D3, bone turnover markers (PTH, P1NP, OC and beta - CTx) and calcium during the two analysed training periods (September 2011, April 2012) in the players participating in the study.

The mean level of 25(OH)D3 was significantly higher after the summer period than after the winter period (30.82 ± 9.04 ng/ml vs 24.96 ± 9.91 ng/ml).

After the summer period, we found that 50% (n = 12) of the players had normal levels of 25(OH)D3, 37.5% (n = 9) had 25(OH)D3 insufficiency and 12.5% (n = 3) had 25(OH)D3 deficiency. The respective values after the winter period were: 16.7% (n = 4), 45.8% (n = 11) and 37.5% (n = 9).

After the summer period, significantly lower PTH levels (21.37±6.88 pg/ml vs 25.37±7.95 pg/ml) and significantly higher calcium levels (2.55±0.08 mmol/l vs 2.46±0.08 mmol/l) were found compared to the measurements taken after the winter period.

In the investigated periods of time, no statistically significant differences in the mean levels of bone turnover markers, i.e. P1NP, OC and beta - CTx, or in the mean values of the OC/beta - CTx ratio were observed.

We found no statistically significant correlations between 25(OH)D3 and body mass composition, calcium or bone turnover markers.

## Discussion

Vitamin D plays an important role in bone metabolism and the normal functioning of the muscular system. The most important causes of decreased levels of vitamin D in the body include insufficient exposure to sunlight and insufficient dietary supply of this vitamin (Holick, 2007).

We found significantly lower levels of 25(OH)D3 in Polish professional soccer players after the winter period compared to the summer period (24.96±9.11 ng/ml vs 30.82±9.04 ng/ml).

Morton et al. (2012) also demonstrated a significant reduction in serum levels of 25(OH)D3 in a group of professional soccer players of the English Premier League at the latitude of 53° N between the summer and the winter period (in August and December). Similarly, a study in Spanish professional soccer players who trained at the latitude of 37° N showed a statistically significant reduction in serum levels of 25(OH)D3 between these two time periods (October and February) (Galan et al., 2012).

It should be noted that 25(OH)D3 levels in our players in September were about 20% lower than those in professional soccer players of the English Premier League (Morton et al., 2012) in August while training at a similar latitude.

Bolland et al. (2007) suggest that changes in vitamin D levels should be interpreted with respect to the changes in fat mass, as they are negatively correlated with the amount of body fat.

Our study failed to show any significant differences in body fat between the investigated periods or any significant correlation between 25(OH)D3 levels and the amount of body fat

It should be emphasised that the literature lacks a unified normal range for serum levels of 25(OH)D3. According to Vieth (2011), the lower limit of the normal physiological level of 25(OH)D3 ranges from 40 to 100 ng/ml. The Institutes of Medicine suggest that the sufficient level of 25(OH)D3 is 20 ng/ml (IOM, 2011). According to the Endocrine Society, normal levels of 25(OH)D3 are 30 – 60 ng/ml, insufficiency is defined as levels of 21 – 29 ng/ml and deficiency is defined as levels below 20 ng/ml (Holick et al., 2011).

In our study, we followed the criteria proposed by the Endocrine Society. It was found that after the summer season (September) 50% of the players had normal levels of 25(OH)D3, 37.5% had 25(OH)D3 insufficiency and 12.5% had 25(OH)D3 deficiency. The respective values after the winter period (April) were: 16.7%, 45.8% and 37.5%. These findings suggest that it might be necessary to monitor 25(OH)D3 levels particularly in the winter period in professional soccer players.

Morton et al. (2012) found that 65% of soccer players had 25(OH)D3 insufficiency (<20 ng/ml) in winter. Galan et al. (2012) showed that after the winter period (early February) 64% of players had 25(OH)D3 levels below 30 ng/ml and only 14.3% had normal levels.

Analyses conducted in various groups of athletes such as runners (Lethonen - Veromaa et al., 1999; Willis et al., 2008), gymnasts (Lethonen - Veromaa et al., 1999; Lovell, 2008) or amateur athletes (Halliday et al., 2011) revealed that 37 – 100% of the subjects had 25(OH)D3 deficiency and 1 – 83% of the subjects had 25(OH)D3 insufficiency. These results depended on the sports discipline, study period and geographic location.

It should be emphasised that 25(OH)D3 levels may significantly affect exercise capabilities of athletes. Multiple studies have shown a considerable correlation of vitamin D levels with muscle structure and strength (Wicherts et al., 2007; Mowe et al., 1999; Houston et al., 2007; Bischoff -Ferrari et al., 2004b; Bischoff et al., 1999). Visser et al. (2003) found that a 25(OH)D3 level of 30 ng/ml guarantees optimal muscle function. Close et al. (2013) suggest that decreased vitamin D levels are one of the important causes of reduced exercise capabilities in athletes. The ergogenic effect of vitamin D may be related to the regulation of muscle protein synthesis thanks to the presence of vitamin D receptors in skeletal myocytes (Bischoff - Ferrari et al., 2004a). This mechanism is not, however, completely elucidated and requires further studies.

We showed significantly higher levels of 25(OH)D3 and calcium as well as lower levels of PTH after the summer period versus the measurements taken after the winter period. These findings are consistent with those of Galan et al. (2012). Decreased serum calcium may stimulate the secretion of PTH, which activates reactions leading to the formation of 1,25(OH)2D2, which may in turn increase calcium absorption from the intestines, decrease renal elimination of calcium and increase bone resorption. We did not, however, observe any correlation between PTH levels and 25(OH)D3 levels.

There are many reports demonstrating that high physical activity affects the levels of bone turnover markers and depends on the type of physical exertion (Maïmoun and Sultan, 2009; Maïmoun and Sultan, 2011). It has also been shown that the reduction in bone mineral density during winter months may be caused by a decreased intake of vitamin D, what causes an increased secretion of PTH (Zofkova and Hill, 2007).

A study of Wolman et al. (2013) in female ballet dancers showed significantly lower levels of 25(OH)D3 and higher levels of P1NP in the winter period (February) compared to the summer period (August), although no changes in CTx were observed.

In our study, the significant reductions in 25(OH)D3 levels after the winter period did not result in any significant changes in the levels of bone turnover markers (P1NP, OC, beta - CTx, OC/beta - CTx). There were also no significant correlations between 25(OH)D3 levels and the levels of bone turnover markers.

Lombardi et al. (2011) reported changes in the levels of bone turnover markers in elite female skiers analysed during a year-long preparation for competitions but found no significant correlation between 25(OH)D3 levels and osteocalcin or CTx levels. They also showed that an increase in the levels of bone turnover markers was more related to training loads than to the changes in vitamin D levels.

To summarise, levels of 25(OH)D3 in Polish professional soccer players after the summer period are higher than those after the winter period, which may first of all be related to lower exposure to UVB. This does not, however affect the levels of bone turnover markers in a significant manner. A slight trend towards reduced levels of bone turnover markers after the winter season compared to the time point after the summer season is, however, noted.

It therefore seems justified to monitor the levels of 25(OH)D3, calcium and PTH in professional soccer players, particularly after the winter period, when the exposure to sunlight is considerably limited.

It also seems that soccer players with decreased 25(OH)D3 concentration should receive a vitamin D – enhanced diet. In case of considerable vitamin D deficit supplementation with oral preparations of vitamin D is necessary (apart from diet modification).

## Figures and Tables

**Table 1:** The mean values of anthropometric variables in soccer players participating in the study after two periods of training: the summer period and the winter period (the values are given as means ± SD)

	September 2011	April 2012
Body mass (kg)	78.71±5.42	79.61±6.71
Body cell mass (kg)	38.31±3.97	39.11±4.34
Total body water (l)	47.04±4.59	47.28±4.81
Extracellular water (l)	19.02±2.14	18.79±2.10
Intracellular water (l)	28.02±2.85	28.49±3.22
Fat mass (kg)	16.91±3.89	15.55±3.18
Fat-free mass (kg)	64.15±6.50	64.50±6.69
Muscle mass (kg)	46.39±4.71	47.24±5.14

**Table 2:** Serum levels of bone turnover markers (mean ± SD) in soccer players during the two investigated periods

	September 2011	April 2012	p value
25(OH)D_3_ (ng/ml)	30.82±9.04	24.96±9.91	0.0003
PTH (pg/ml)	21.37±6.83	25.37±7.95	0.0062
P1NP (ng/ml)	81.78±30.24	79.83±22.57	NS
OC (ng/ml)	35.26±12.60	33.00±9.05	NS
Beta - CTx (ng/ml)	0.71±0.21	0.68±0.22	NS
OC/beta - CTx	50.97 ± 15.04	50.60 ± 11.34	NS
Calcium (mmol/l)	2.55±0.08	2.46±0.08	0.0004

## References

[b1-jhk-38-135] Bischoff HA, Stähelin HB, Urscheler N, Ehrsam R, Vonthein R, Perrig-Chiello P, Tyndall A, Theiler R (1999). Muscle strength in the elderly: its relation to vitamin D metabolites. Arch Phys Med Rehabil.

[b2-jhk-38-135] Bischoff - Ferrari HA, Borchers M, Gudat F, Dürmüller U, Stähelin HB, Dick W (2004a). Vitamin D receptor expression in human muscle tissue decreases with age. J Bone Miner Res.

[b3-jhk-38-135] Bischoff - Ferrari HA, Dietrich T, Orav EJ, Hu FB, Zhang Y, Karlson EW, Dawson - Hughes B (2004b). Higher 25 -hydroxyvitamin D concentrations are associated with better lower - extremity function in both active and inactive persons aged > or =60 y. Am J Clin Nutr.

[b4-jhk-38-135] Bolland MJ, Grey A, Cundy T, Reid IR (2007). Defining vitamin D deficiency. N Z Med J.

[b5-jhk-38-135] Cannell JJ, Hollis BW, Sorenson MB, Taft TN, Anderson JJ (2009). Athletic performance and vitamin D. Med Sci Sport Exer.

[b6-jhk-38-135] Close GL, Leckey J, Patterson M, Bradley W, Owens DJ, Fraser WD, Morton JP (2013). The effects of vitamin D3 supplementation on serum total 25[OH]D concentration and physical performance: a randomised dose - response study. Br J Sports Med.

[b7-jhk-38-135] Galan F, Ribas J, Sánchez - Martinez PM, Calero T, Sánchez AB, Muñoz A (2012). Serum 25 - hydroxyvitamin D in early autumn to ensure vitamin D sufficiency in mid - winter in professional football players. Clin Nutr.

[b8-jhk-38-135] Halliday TM, Peterson NJ, Thomas JJ, Kleppinger K, Hollis BW, Larson - Meyer DE (2011). Vitamin D status relative to diet, lifestyle, injury, and illness in college athletes. Med Sci Sports Exerc.

[b9-jhk-38-135] Hamilton B (2010). Vitamin D and human skeletal muscle. Scand J Med Sci Sports.

[b10-jhk-38-135] Holick MF (2007). Vitamin D deficiency. New Engl J Med.

[b11-jhk-38-135] Holick MF, Binkley NC, Bischoff - Ferrari HA, Gordon CM, Hanley DA, Heaney RP, Murad MH, Weaver CM, Endocrine Society (2011). Evaluation, treatment, and prevention of vitamin D deficiency: an Endocrine Society clinical practice guideline. J Clin Endocrinol Metab.

[b12-jhk-38-135] Houston DK, Cesari M, Ferrucci L, Cherubini A, Maggio D, Bartali B, Johnson MA, Schwartz GG, Kritchevsky SB (2007). Association between vitamin D status and physical performance: The InCHIANTI Study. J Gerontol A Biol Sci Med Sci.

[b13-jhk-38-135] Institutes of Medicine (2011). Dietary reference intakes for calcium and vitamin D.

[b14-jhk-38-135] Knaysi GA, Crikelair GF, Cosman B (1968). The role of nines: its history and accuracy. Plast Reconstr Surg.

[b15-jhk-38-135] Lehtonen - Veromaa M, Möttönen T, Irjala K, Kärkkäinen M, Lamberg-Allardt C, Hakola P, Viikari J (1999). Vitamin D intake is low and hypovitaminosis D common in healthy 9- to 15-year-old Finnish girls. Eur J Clin Nutr.

[b16-jhk-38-135] Lombardi G, Colombini A, Freschi M, Tavana R, Banfi G (2011). Seasonal variation of bone turnover markers in top-level female skiers. Eur J Appl Physiol.

[b17-jhk-38-135] Lovell G (2008). Vitamin D status of females in an elite gymnastics program. Clin J Sport Med.

[b18-jhk-38-135] Maïmoun L, Sultan C (2009). Effect of physical activity on calcium homeostasis and calciotropic hormones: a review. Calcif Tissue Int.

[b19-jhk-38-135] Maïmoun L, Sultan C (2011). Effects of physical activity on bone remodeling. Metabolism.

[b20-jhk-38-135] Morton JP, Iqbal Z, Drust B, Burgess D, Close GL, Brukner PD (2012). Seasonal variation in vitamin D status in professional soccer players of the English Premier League. Appl Physiol Nutr Metab.

[b21-jhk-38-135] Mowe M, Haug E, Bohmer T (1999). Low serum calcidiol concentration in older adults with reduced muscular function. J Am Geriatr Soc.

[b22-jhk-38-135] Vieth R (2011). Why the minimum desirable serum 25 - hydroxyvitamin D level should be 75 nmol/L (30 ng/ml). Best Pract Res Clin Endocrinol Metab.

[b23-jhk-38-135] Visser M, Deeg D, Lips P (2003). Low Vitamin D and high parathyroid hormone levels asdeterminants of loss of muscle strength and muscle mass (Sarcopenia): The Longitudinal Aging Study Amsterdam. J Clin Endocr Metab.

[b24-jhk-38-135] Wicherts IS, van Schoor NM, Boeke AJ, Visser M, Deeg DJ, Smit J, Knol DL, Lips P (2007). Vitamin D status predicts physical performance and its decline in older persons. J Clin Endocr Metab.

[b25-jhk-38-135] Willis KS, Peterson NJ, Larson – Meyer DE (2008). Should we be concerned about the vitamin D status of athletes?. Int J Sport Nutr Exerc Metab.

[b26-jhk-38-135] Wolman R, Wyon MA, Koutedakis Y, Nevill AM, Eastell R, Allen N (2013). Vitamin D status in professional ballet dancers: Winter vs. summer. J Sci Med Sport.

[b27-jhk-38-135] Zofkova I, Hill M (2007). Long-term 1,25(OH)2 vitamin D therapy increases bone mineral density in osteopenic women. Comparison with the effect of plain vitamin D. Aging Clin Exp Res.

